# The Ethanolic Extract of *Eysenhardtia polystachya* (Ort.) Sarg. Bark and Its Fractions Delay the Progression of Rheumatoid Arthritis and Show Antinociceptive Activity in Murine Models

**Published:** 2018

**Authors:** Saudy Saret Pablo-Pérez, Benjamín Parada-Cruz, Olivier Christophe Barbier, María Estela Meléndez-Camargo

**Affiliations:** a *Departamento de Farmacia, Escuela Nacional de Ciencias Biológicas del Instituto Politécnico Nacional (ENCB-IPN), Mexico City, 07738, México. *; b *Departamento de Toxicología, Centro de Investigación y de Estudios Avanzados del Instituto Politécnico Nacional (CINVESTAV-IPN), Mexico City, 07360, Mexico.*

**Keywords:** *Eysenhardtia polystachya*, Rheumatoid arthritis, Antinociceptive, Complete Freund´s adjuvant, Cytokines

## Abstract

*Eysenhardtia polystachya *is widely used in folk medicine as an anti-rheumatic and analgesic agent, but no systematic study of its effects on several markers associated with rheumatoid arthritis and its ethnomedical use as analgesic agent has been performed. We evaluated the anti-arthritic and antinociceptive properties of an ethanolic extract of *E. polystachya* (EE) bark and its rich-flavonoids fractions in murine models. The EE was administered orally at doses of 25, 50, 100, and 200 mg/kg/day, and its fractions at 25 mg/kg/day in all animal models. Anti-arthritic activity was evaluated using a complete Freund´s adjuvant (CFA)-induced rheumatoid arthritis model in rats. The severity of arthritis was evaluated by changes in paw oedema, body weight, arthritic index, radiological scores, histological assessment of synovial joints, erythrocyte sedimentation rate, haematocrit, haemoglobin, serum rheumatoid factor, serum C-reactive protein and serum levels of the pro-inflammatory cytokines IL-1β, IL-6, TNF-α, IL-18, IFN-γ, GM-CSF, and anti-inflammatory cytokines IL-4, IL-10, IL-13. Antinociceptive activity was evaluated using an acetic acid-induced abdominal contraction test and a hot-plate test in mice. EE and its rich-flavonoids fractions inhibited secondary inflammatory reactions, diminished the specific histopathological alterations in the joint capsule and reduced the serum concentrations of the pro-inflammatory cytokines IL-6, TNF-α, and GM-CSF in arthritic rats. EE also reduced the number of writhes produced by acetic acid and increased the response time on the hot plate for mice. Our findings support the use of *Eysenhardtia polystachya* bark for the treatment of rheumatoid arthritis and pain management.

## Introduction

Rheumatoid arthritis (RA) is a systemic, autoimmune and chronic inflammatory disease that is associated with long-term disability, cachexia, anaemia, and premature mortality (1-4). Many different pathways contribute to the pathogenesis of RA, especially pro-inflammatory cytokine pathways such as those mediated by tumour-necrosis factor alpha (TNF-α), interleukin 1 beta (IL-1β), IL-18, IL-6, interferon gamma (IFN-γ), and granulocyte-macrophage colony-stimulating factor (GM-CSF). IL-1, IL-6, and TNF-α are the key cytokines that drive inflammation in RA (5). These cytokines activate cells in their local environments and continue the production of cytokines; this in turn creates a positive feedback loop between fibroblasts and macrophage-like synoviocytes, perpetuating synovial inflammation (6). Moreover, these soluble pro-inflammatory molecules activate various signal transduction cascades and activate transcription factors, which subsequently induce the over-expression of matrix metalloproteinases (MMPs), osteoclast formation, and synovial proliferation, ultimately leading to the destruction of joints and functional impairments (4, 6 and 7). 

Currently, analgesics, non-steroidal anti-inflammatory drugs (NSAIDs), steroids, immunosuppressant drugs and disease-modifying anti-rheumatic drugs including TNF-α or IL-1 blockade are recommended to reduce pain and inflammation, halt disease progression, minimize disability and improve the quality of life of patients (2). However, various adverse effects such as gastrointestinal disorders, cardiovascular risks and humoral immune disorders, as well as high costs limit the effectiveness of these drugs, and most patients discontinue treatment. For this reason, new and more effective therapeutic agents that could be used for long periods, at a lower cost and with fewer side effects are required (4, 7 and 8). Several phenols have shown analgesic and anti-inflammatory activity, as well as the ability to limit the degradation of cartilage *in-vitro* and maintain joint structure in animal models of RA. Thus, many natural products, such as flavonoids, may modulate the inflammatory response without side effects, constituting “gold mines” for treatment of RA (9, 10). 

*Eysenhardtia polystachya *(Ort.) Sarg., is a small tree native to Mexico in the family Leguminosae that grows wild and abundantly in fields. It is commonly known as “palo azul” (blue wood) or “palo dulce” (sweet wood) (11). Phytochemical studies indicate that *E. polystachya* contains polyphenols, and previous chemical examination of this species led to the isolation and structural elucidation of several flavonoids (11, 12). *E. polystachya *is widely used in folk medicine as a blood depurative, antitussive, antispasmodic, antidiabetic, febrifuge, anti-inflammatory, anti-rheumatic, and analgesic agent (12). Published reports indicate that the aqueous extract of the bark of *E. polystachya *prevents the formation of kidney stones *in-vivo* (13). 

The methanol-water extract showed antidiabetic and anti-hyperlipidemic activities, an ability to reduce the formation of advanced glycation end products, and an antioxidant capacity *in-vitro* (12). However, a survey of the literature showed that no systematic study of its effects on several markers associated with rheumatoid arthritis and its ethnomedical use as analgesic agent has been performed. With this focus, we assessed the anti-arthritic and antinociceptive activity of the ethanol extract of the bark of *Eysenhardtia polystachya *and its rich-flavonoids fractions to clarify the traditional use of the plant in Mexican folk medicine. 

## Experimental

*Reagents and drugs*

Analytical-grade reagents were used. Acetylsalicylic acid, aluminium chloride, complete Freund’s adjuvant and indomethacin were obtained from Sigma Chemical Co. (USA). Acetic acid and sodium bicarbonate were obtained from J.T. Baker (Mexico). Quercetin was obtained from Fluka (Switzerland).

*Plant material*

*Eysenhardtia polystachya* (Ort.) Sarg. was collected with the permission of the Mexican authorities in San Pedro Tlaquilpan, Zempoala (19° 55’ N, 98° 40’ W), State of Hidalgo, Mexico, in February, 2010. The botanical identiﬁcation and authentication of the plant samples was performed by Biologist Laura Doval Ugalde at Escuela Nacional de Ciencias Biológicas of Instituto Politécnico Nacional (ENCB-IPN). The plant samples were compared to a voucher specimen deposited in the ENCB herbarium under number 247. The bark was air-dried at room temperature in the shade, and then was ground in a mill (FITZ^®^ MILL model D Comminutor, Industrial Drive Elmhorst, 

USA).

*Preparation and fractionation of the plant extract*

Approximately 6,100 g of the dried and powdered bark of *Eysenhardtia polystachya* was extracted exhaustively with 16.5 L of ethanol by maceration. The ethanolic extract (EE) was filtered, and the solvent was evaporated *in-vacuo* at 40 °C in a rotary evaporator (Büchi R-124, Switzerland). 

The EE was fractionated by column chromatography (CC) using silica gel (230-400 mesh), eluting with a mixture of ethyl acetate/methanol/water(70:25:25, v/v). Twenty-six fractions were obtained and after various analyses by thin layer chromatography on silica gel F_254_ and phytochemical tests were grouped into five major fractions (F1-F5).

*Phytochemical screening*

The secondary metabolites of the EE used in this study were detected by standard colour and precipitation phytochemical tests. Briefly, Dragendorff´s reaction was used to characterize alkaloids, Fehling´s and Benedict´s reactions for reducing sugars, Erlich´s reaction and observation under UV light of alkalinized extracts for coumarins, Kedde, Legal´s and Baljet´s reactions for cardiac glycosides, a frothing test for saponins, a FeCl_3 _test for tannins, Liebermann-Buchard´s reaction for triterpenoids and steroids, Bornträger’s reaction for quinones and Shinoda´s reaction for flavonoids, among others (14, 15).

*Determination of total flavonoid content*

The amount of total flavonoids in the EE and its fractions was measured by a colorimetric assay according to the method of Lamaison and Carnet (16), with some modifications. 

An aliquot (0.8 mL) of appropriately diluted sample solution was added to test tubes, and 100 μL of 10% (w/v) AlCl_3 _solution was added with mixing. The absorbance of the mixture was determined at 440 nm using a spectrophotometer (Cary 50 probe, Varian, Australia).

Quantification was performed on the basis of a standard curve prepared using quercetin, and the results are expressed as milligrams of quercetin equivalent (QE) per gram of 

extract.

*Preparation of the test samples for the bioassay*

To evaluate the anti-arthritic and antinociceptive effects of *E. polystachya*, the EE was reconstituted in water and administered at doses of 25 mg/kg (EE 25), 50 mg/kg (EE 50), 100 mg/kg (EE 100), and 200 mg/kg (EE 200) body weight (bw). The fractions F1-F5 of EE were also dissolved in water, except for F1 that was dissolved in peanut oil (PO control). All fractions of the EE were administered at a dose of 25 mg/kg bw. The animals in the control groups received the same experimental handling as the test groups, except that drug treatment that was replaced with an appropriate volume of vehicle. Either 2 or 10 mg/kg bw of indomethacin (IND 2 and IND 10, respectively) or 100 mg/kg bw of acetylsalicylic acid (ASA 100) in 5% sodium bicarbonate (SB control) were used as reference drugs. In all groups, the administration was *per os* and there were at least six animals per group.

*Animal care and management*

Adult female Wistar rats (180-220 g) and female NIH mice (20-30 g) were used. They were housed and maintained in the animal house at room temperature (22–24 °C) and 50–55% relative humidity, with day/night cycles of 12 × 12 h. The animals were fed a standard rodent diet with water *ad libitum*. Care and handling of animals followed international accepted procedures according to the Institute for Laboratory Animal Research’s Guide for Care and Use of Laboratory Animals. 

*Complete Freund’s adjuvant-induced rheumatoid arthritis*

A model of RA induced by complete Freund’s adjuvant (CFA), according to established protocols (17) was used with minor modifications. On day 0, two groups of rats were given a single subcutaneous injection in the footpad of the left metatarsal hind limb as follows: 1, the arthritic group received 100 µL of CFA (1 mg/mL of *Mycobacterium tuberculosis* in 85% paraffin oil and 15% mannide monooleate) and 2, the normal control group received 100 µL of SSI 0.9%. At 14 days post-CFA injection, arthritic rats were divided into twelve groups and were treated with the EE 25, EE 50, EE 100, EE 200, and the fractions F1 25, F2 25, F3 25, F4 25 and F5 25. Animals in the control groups received SB or PO, and the reference group received IND 2. The treatments were orally administered for 7 days.

*Assessment of arthritis score in the adjuvant induced arthritis rats*

The severity of the inflammatory response was graded by an arthritis score. The score was determined by considering oedema and erythema of the toes, paws and ankles of all arthritic rats on a scale of 0-4, where 0, no erythema or swelling; 1, erythema and swelling of toes; 2, swelling of paws; 3, swelling of ankles; and 4, ankylosis, inability to move the whole leg (7, 10). The score was recorded on 14^th^, 16^th^, 18^th^, 20^th^ and 21^st^ days post-CFA injection. Furthermore, changes in the thickness and width of the paws and the rear ankles (left and right) were measured with a micrometer (Mitutoyo Co., Japan), while body weight was also measured as an additional parameter of the severity of arthritis (8).

*Assessment of biochemical parameters*

On day 0 before the induction of RA, day 14 prior to the administration of treatments and day 21 before the slaughter of animals, blood samples from each rat were obtained by puncture of the retro-orbital plexus. Part of this blood was used to determine the erythrocyte sedimentation rate (ESR), which was measured by the Westergren method (15) with some modifications and expressed as mm of plasma cleaned per h. The haematocrit was determined and read as the percent of whole venous blood occupied by red blood cells (RBCs). 

The haemoglobin concentration (g/dL) in the blood was determined by the cyanmethaemoglobin method with a commercial kit (Randox Laboratories, UK). All blood samples were tested in parallel.

Another part of the collected blood was allowed to stand 30 min at room temperature for coagulation. Serum was separated by centrifugation at 15,000×g for 10 min and stored at -20 °C until use. Rheumatoid factor (RF) and C-reactive protein (CRP) were measured by the agglutination method with commercial kits (Randox Laboratories, UK).

*Quantification of cytokines in serum*

A magnetic bead panel of rat cytokines/chemokines (Millipore Corp., USA) was used to quantify the pro-inflammatory cytokines IL-1β, IL-6, TNF-α, the IL-18, IFN-γ and GM-CSF and the anti-inflammatory cytokines IL-4, IL-10 and IL-13. We followed the manufacturer’s instructions. This pre-validated assay is based on Luminex® xMAP® technology, which is capable of performing a variety of immunoassays on the surface of fluorescence-coded magnetic beads. 

The serum samples were gradually thawed approximately 1.5 h before the test to room temperature (20-25 °C), stirred, and centrifuged to remove particles. Twenty-five µL of each serum sample was diluted 1:2 with the shock absorber provided in the kit. High quality controls were included at low concentrations. The plate was run on a MAGPIX® (Merck-Millipore, USA). Cytokine concentrations are expressed as pg/mL serum.

*Radiographic and histopathological analysis of synovial joints*

On the day of sacrifice, the hind limb of each rat was amputated at the level of the ankle joint. The samples were fixed in 10% formalin and X-ray images were taken (10) to observe the probable destruction and loss of joint space. Subsequently, the specimens were decalcified with nitric acid, dehydrated and processed for haematoxylin-eosin staining. Safranin O was used to observe the glycosaminoglycans (GAGs) in the extracellular matrix of articular cartilage. Periodic acid-Schiff (PAS) was used to differentiate cells and glycoproteins containing them, such as fibroblasts, fibrocytes, and chondrocytes (15). 

We assessed synovial lining cell hyperplasia, *pannus* formation and fibrosis a using a semi-quantitative grading scale of 0 to 3. Inflammatory cell infiltration, cartilage degradation and bone erosion were graded from 0 to 5. A grade of 0 corresponds to the absence and 3, 4 or 5 to a severe degree of pathological alteration. The sum of all the histological parameter scores was designated as the “histological score” (6).

*Antinociceptive activity in mice*

The ‘hot-plate’ (thermal) and ‘acetic acid’ (chemical) analgesic test methods were used. The experiments were conducted according to the ethical guide for the study of experimental pain in conscious animals (18). The mice were fasted overnight with water given *ad libitum *and then were formed into twelve random treatment groups. The groups of animals were pre-treated orally; in the acetic acid-induced abdominal constriction test the reference group received IND 10 and in the hot plate test they received ASA 100. EE 25, EE 50, EE 100, EE 200 and the fractions F1 25, F2 25, F3 25, F4 25 and F5 25 were evaluated. Animals in the control groups received SB or PO. 

*Acetic acid-induced abdominal constriction test*

The abdominal constriction test described by Koster *et al*. (19) was used with slight modifications. According to the method, 30 min after the administration of test samples, the mice were intraperitoneally injected with 0.1 mL/10 g bw of 0.6% (v/v) acetic acid solution in distilled water to cause a typical stretching response. The mice were kept in individual cages for observation and the total number of abdominal contractions (writhing movements or full extension of both hind paws) was counted for 20 min, starting on the 5^th^ min after the acetic acid injection. In addition, the analgesic effect was measured by calculating the mean reduction in the number of abdominal constrictions for each treatment compared to the SB control. Percent inhibition of writhing was calculated by using the following formula: 

$\mathrm{\% protection}=\left(1-\frac{\mathrm{mean number of writhes}(\mathrm{test})}{\mathrm{mean number of writhes}(\mathrm{control})}\right)\times \mathrm{100}$

 Equation 1

*Hot plate test*

The ‘hot-plate’ (thermal) analgesic test method employed in this study was modified from Eddy and Leimback (20). The temperature was maintained at 55 ± 1 °C. Each mouse was placed on the hot-plate 24 h prior to the experiment to obtain its basal response time (RT_0_) to an electrical heat induced nociceptive pain stimulus. Licking of the paws or jumping was taken as an indicator of the nociceptive response time (RT). Each mouse served as its own control. The day of the experiment, the RT of each mouse was again evaluated at 1, 2, 3 and 4 h after treatment administration. The ability of treatments to slow the RT indicates antinociceptive activity. The percentage of protection against thermal stimuli at each time point was calculated by applying the following formula: 

$\mathrm{\% protection}=\left(\frac{\mathrm{mean RT}(\mathrm{test})}{\mathrm{mean RT}(\mathrm{control})}-1\right)\times \mathrm{100}$

Equation 2

*Acute toxicity study*

An acute toxicity study was performed in mice. The mice were fasted overnight with water given *ad libitum *and randomly assigned into five treatment groups. Four animals were used for complete evaluation at each dose level. The EE at doses from 4, 8, 16 and 32 g/kg bw were administered *per os*. Water was administered to the control group. All deviations in general behaviour associated with the administration of EE were monitored and recorded continuously for the first 3 h after the administration. For the next 14 days, the number of dead animals was also recorded. During this period the animals had access to food and water *ad libitum *(21).

*Statistical analysis*

All data are expressed as the means ± SEM (standard error of mean). To perform statistical analyses, SigmaPlot® 11.0 software was used. To determine significant differences in models of RA and the hot plate, we performed two way repeated measures analysis of variances (RM ANOVA´s). For the rest of tests, we performed one-way ANOVAs. The statistical test Student-Newman-Keuls (S-N-K) was used for *post-hoc* comparisons. Significant differences were set at *P*-values less than 0.05. 

## Results

*Ethanolic extract yield and phytochemical screening and determination of total flavonoids content*

The EE was a brown syrupy consistency; the yield was 5.5% (w/w). Phytochemical screening revealed the presence of medicinally active constituents in the EE as alkaloids, anthraquinones, cardiac glycosides, coumarins, reducing sugars, saponins and tannins. The total flavonoid content of EE and its fractions is shown in Table 1.

**Figure 1:**
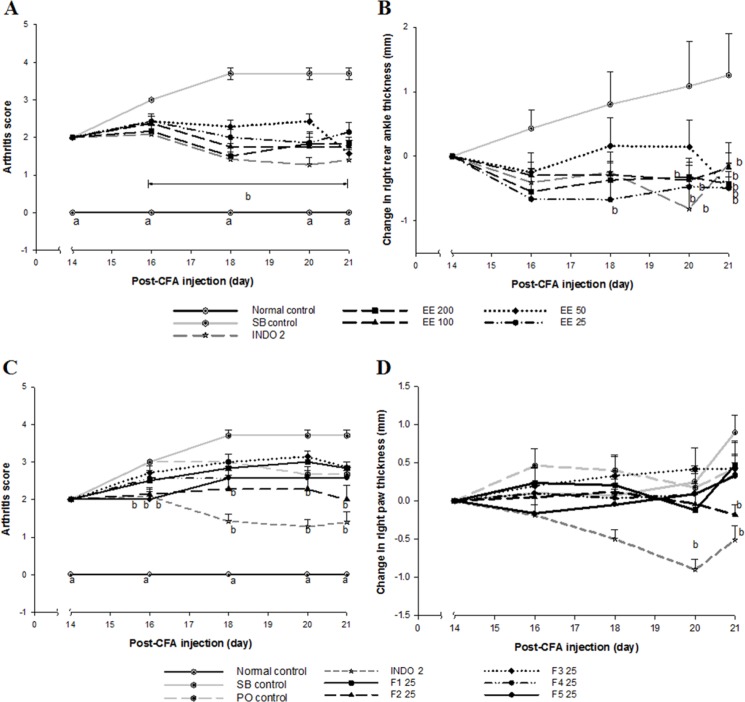
Effect of *Eysenhardtia polystachya *bark (A, C) on arthritic scores and (B) on changes in the thickness of the right rear ankles and (D) on changes in thickness of the right paws in CFA-induced arthritis. Mean ± SEM of at least six animals per group. ^a^*P *< 0.05 compared with the rest of the groups and ^b^*P *< 0.05 compared with the sodium bicarbonate (SB) control group at the same time point

*Effect of Eysenhardtia polystachya on complete Freund’s adjuvant induced arthritis*CFA administration caused chronic inflammation in the left legs (primary lesion) of all rats from day 6 post-injection. Swelling and erythema gradually spread to all four limbs (secondary lesion), the nose, and the base of the tail and ears, leading to symmetrical RA. The prevalence of secondary lesions was approximately 10% of diseased rats. However, erythema and swelling in rats treated with INDO 2, as well as all doses of the EE (Figure 1A) and its fractions (Figure 1C) were reduced (*P *< 0.05) compared with the SB control group. All doses of the EE, F2 and INDO 2 suppressed (*P *< 0.05) secondary inflammation in the CFA-non-injected paw, as shown in Figure 1B and Figure 1D. The arthritic process and treatments did not affect body weight in arthritic rats compared to controls (data not shown).

*Assessment of biochemical parameters*

At 14 days post-CFA injection, ESR was increased (*P < *0.05) in the arthritic group, with value of 12.44 ± 2.48 mm/h compared with a normal control value of 7.11 ± 0.70 mm/h. Haematocrit was decreased (*P < *0.05) in the arthritic group, with a value of 46 ± 1.23% compared with a normal control value of 54 ± 0.76%. Furthermore, serum from arthritic rats tested positive for RF and CRP compared with normal control rat serum that tested negative. The haemoglobin concentration was not altered (data not shown). Twenty-one days post-CFA injection at the end of the treatment period, values of ESR, haematocrit and haemoglobin were not altered (data not shown) and serum from arthritic rats continued to be positive for RF and CRP.

*Quantification of cytokines in serum*

**Figure 2:**
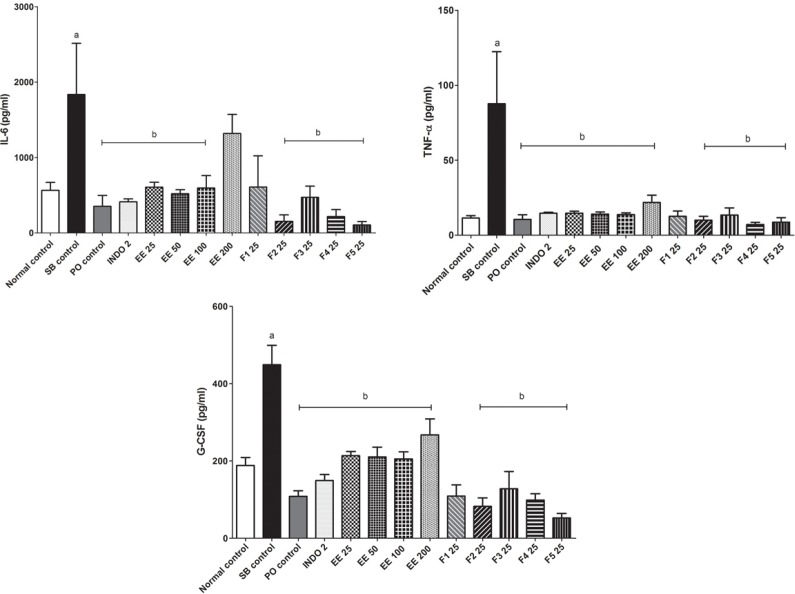
Effect of *Eysenhardtia polystachya *on serum IL-6, TNF-α and G-CSF concentrations in CFA-induced arthritis. Mean ± SEM of six replicates. ^a^*P < 0*.05 compared with the normal control group and ^b^*P < 0*.05 compared with the sodium bicarbonate (SB) control group

The serum concentration of the pro-inflammatory cytokines IL-6, TNF-α, and GM-CSF was increased (*P* < 0.05) in the SB control group with respect to the control group. After treatment for 7 days with the EE and its fractions, except F1 25, the concentration of pro-inflammatory cytokines IL-6, TNF-α and GM-CSF decreased (*P < *0.05) compared with the SB control group (Figure 2). Serum concentrations of the cytokines IL-1β, IFN-γ and IL-18 did not increase with the CFA induced arthritic process after 21 days, and treatments did not induce changes (data not shown). Serum concentrations of anti-inflammatory cytokines IL-13, IL-10 and IL-4 in all groups were similar (data not shown).

*Radiographic and histopathological analysis of synovial joints*

**Figure 3:**
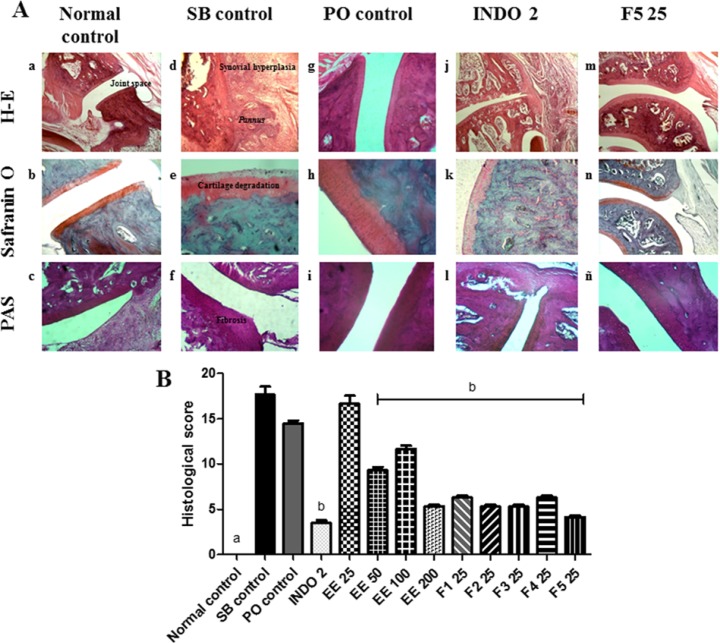
A) Histological assessment of the effect of *Eysenhardtia polystachya *on the synovial joint. (a-c) normal synovial joint architecture. (j-l) minimal synovitis with primarily mononuclear inflammatory cell infiltration in the synovial membrane, *pannus* formation and a minimal degeneration of cartilage. (m-ñ) average synovitis with increased infiltration of mononuclear inflammatory cells in the synovial membrane, minimum* pannus* formation, cartilage degeneration and fibrosis. (g-i) proliferation and infiltration of a large number of mononuclear cells, minimal erosion of the subchondral bone, cartilage damage and surface fibrosis. (d-f) severe destruction of cartilage and subchondral bone, thickening fibres and severe fibrosis, bony ankylosis. (B) Effect of *Eysenhardtia polystachya* on the histological scores of synovial joint damage caused by rheumatoid arthritis. Mean ± SEM of six replicates. ^a^*P < 0*.05 compared with the rest of the groups, ^b^*P < 0*.05 compared with the respective control groups

No apparent radiographic changes in the bones and synovial joints in any of the arthritic groups were observed compared to the normal control group. We observed a radiopaque zone around the patellar tibiofibular joint, primarily in the left leg, which corresponded to the area of soft tissue inflammation (data not shown). In histopathological analysis, typical features of RA were observed in the SB control group compared with the normal control group, which included infiltration of inflammatory cells, GAGs depletion, *pannus *formation and thickened fibres in articular cartilage and bony ankylosis (Figure 3A:a-f). In the PO control group, the damage was less severe than in the SB control group (Figure 3A:g-i). In the INDO 2 group, proliferation and infiltration of mononuclear cells and the *pannus* were partially inhibited, the destruction of articular cartilage was lower than in the SB control group, and no bone erosion was found (Figure 3A:j-l). In F5 25 treated rats, the typical histology of RA was markedly reduced (Figure 3A:m-ñ). The EE 50, EE 100, EE 200 and remaining fractions of EE also partly inhibited the damage produced by AR. Although proliferation and infiltration of mononuclear cells, the *pannus*, mild cartilage destruction articular and fibrosis were observed, no bone erosion was found (Figure 3B).

*Antinociceptive activity of Eysenhardtia polystachya in mice*

*Acetic acid-induced abdominal constriction test*

**Figure 4:**
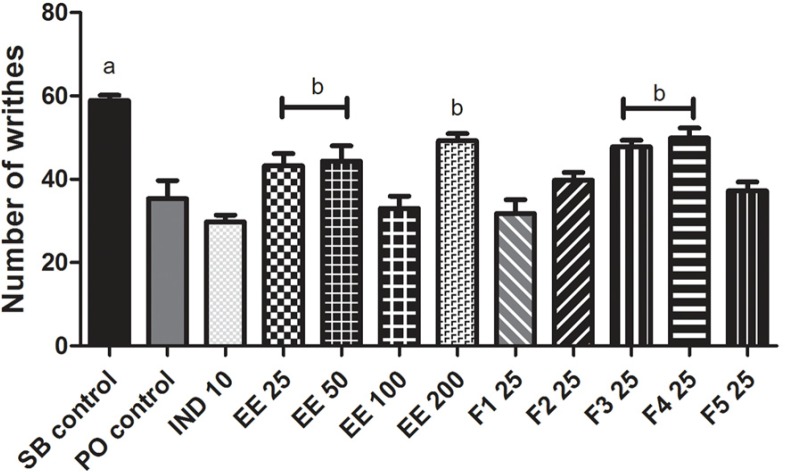
Effect of the ethanolic extract of *Eysenhardtia polystachya *bark and its fractions on acetic acid-induced writhing in mice. Mean ± SEM of eight animals per group. ^a^*P < 0*.05 compared with the rest of the groups, ^b^*P < 0*.05 compared with the indomethacin group

The number of writhes produced by acetic acid in mice previously treated with IND 10 decreased by 49% compared with the SB control group. The EE 25, EE 50, EE 100 and EE 200 reduced mean writhing by 27, 25, 44 and 16%, respectively, compared with the SB control group. F2, F3, F4 and F5 (25 mg/kg bw) also reduced the number of writhes by 32, 19, 15 and 37%, respectively, compared with the SB control group. The PO control group decreased the number of writhes by 40% compared with the SB control group, but the fraction F1 had no antinociceptive effect because it only reduced writhing by 10% compared with the PO control group. The EE 100, F2 and F5 fractions induced a similar antinociceptive effect to indomethacin (Figure 4).

*Hot plate test*

The RT against a thermal pain stimulus in the control groups remained unchanged during the study. In the group treated with ASA 100, an increase (*P *< 0.05) was observed in the RT during the evaluation period, with maximum protection in the 3^rd^ and 4^th^ h at a percentage of protection of 58 and 44%, respectively. Only the dose of 200 mg/kg of EE increased (*P *< 0.05) the RT in the 4^th^ h with respect to the SB control group. Fractions F1, F2, and F5 increased (*P *< 0.05) the RT in the 1^st^, 2^nd^ and 4^th^ h after administration compared with the respective control group (Table 2).

*Acute toxicity*

None of the doses of EE produced mortality. Moreover, any signs of toxicity were observed after oral administration. Because no deaths were observed at the dose levels used, the LD_50_ was assumed to be greater than 32 g/kg.

## Discussion

Pain and inflammation are some of the most common manifestations of many diseases that afflict millions of people worldwide, including RA. Although there are drugs used to relieve these symptoms, many people in developing countries continue to use herbal medicine for the treatment of various diseases, as they provide relief from their symptoms and produce fewer side effects than allopathic treatments (22). 

Many plant constituents, including flavonoids, have proven effective against arthritis by reducing cartilage degradation, diminishing leukocyte infiltration in the synovial space, decreasing serum cytokine levels, and other mechanisms (9). Thus, interest in alternative treatments and herbal therapies has intensified among arthritis patients, although there is a lack of scientific validation (10). Flavonoids of numerous plant species have shown anti-inflammatory activity *in-vitro* and *in-vivo*, and several mechanisms of action have been proposed to explain the *in-vivo* pharmacological actions, although the mechanisms are still not fully understood (23).

The quantification of flavonoids in natural products is becoming more common because these data allow the identification of flavonoids with high pharmacological potential. Flavones and flavonols (3, 5-hydroxyflavones and 3, 5-hidroxiflavonoles) form stable yellow complexes with AlCl_3_ and can be analysed by UV/Vis spectrophotometry (24). The results of our quantification showed that *Eysenhardtia polystachya *is a rich source of flavonoids, and that F4 contained the most flavonoids compared with the rest of the fractions.

CFA induced RA is one of the most used models to study the anti-inflammatory and anti-rheumatic properties of various compounds (1, 2, 3, 6, 7 and 14) because it produces many clinical and immunological characteristics similar to human RA. The injection of CFA induces systemic inflammation attributed to the cell wall component of the mycobacterium, muramyl dipeptide, which causes a nonspecific cellular immune response and enhances the production of certain immunoglobulins (1, 2 and 6). The appearance of lesions in the joints of rats after the introduction of bacterially derived antigens implies that CFA cross-reacts with a tissue antigen located in the joint. Recently, it was determined that the pathogenic immunizing antigen of CFA is the mycobacterial heat shock protein mHsp65, suggesting a possible case of antigenic mimicry (6, 17).

**Table 1:** Total flavonoid content of the ethanolic extract of *Eysenhardtia polystachya *bark and its fractions.

**Sample**	**Total flavonoid content** **(mg of EQ/g of** **extract)**
EE	3.72 ± 0.64
F1	2.91 ± 0.03
F2	6.19 ± 0.10
F3	3.28 ± 0.16
F4	9.20 ± 0.05
F5	1.23 ± 0.11^a^

Values are the means ± SEM of three replications. Total flavonoid content is expressed as mg of quercetin equivalents (QE/g of extract).

a *P *< 0.05 compared with F4.

**Table 2:** Effect of the ethanolic extract of *Eysenhardtia polystachya* bark and its fractions on hot-plate induced nociception in mice

**Treatment group **	**Response time (s), (% protection)**				
	**0 h**	**1 h**	**2 h**	**3 h**	**4 h**
SB control	1.21 ± 0.12	1.32 ± 0.13	1.28 ± 0.12	1.42 ± 0.19	1.32 ± 0.18
PO control	1.11 ± 0.09	1.16 ± 0.10	1.23 ± 0.07	2.09 ± 0.10	2.28 ± 0.22
ASA 100	1.23 ± 0.15	1.23 ± 0.11 (-7)	1.60 ± 0.17 (25)	2.24 ± 0.30^a^ (58)	1.89 ± 0.20^a^ (44)
EE 25	1.24 ± 0.11	1.43 ± 0.20 (8)	1.16 ± 0.11 (-9)	1.48 ± 0.23 (4)	1.68 ± 0.24 (27)
EE 50	1.31 ± 0.16	1.35 ± 0.24 (2)	1.69 ± 0.23 (32)	1.78 ± 0.17 (25)	1.22 ± 0.20 (-7)
EE 100	1.29 ± 0.11	0.17 ± 0.08 (-11)	1.51 ± 0.16 (18)	1.44 ± 0.19 (2)	1.29 ± 0.11 (-2)
EE 200	1.25 ± 0.21	1.35 ± 0.15 (2)	1.45 ± 0.19 (13)	1.24 ± 0.15 (-13)	2.11 ± 0.50^a^ (60)
F1 25	1.21 ± 0.14	1.84 ± 0.31^b^ (59)	3.15 ± 0.23b (155)	1.79 ± 0.12 (-14)	3.29 ± 0.55^b^ (44)
F2 25	1.53 ± 0.12	1.86 ± 0.26 (41)	2.47 ± 0.34^a^ (94)	1.84 ± 0.26 (29)	2.44 ± 0.46^a^ (85)
F3 25	1.20 ± 0.09	1.57 ± 0.20 (19)	1.07 ± 0.07 (-16)	1.94 ± 0.30 (36)	1.39 ± 0.20 (6)
F4 25	1.13 ± 0.10	1.07 ± 0.13 (-19)	1.27 ± 0.07 (-1)	1.41 ± 0.21 (-1)	1.04 ± 0.15 (-21)
F5 25	1.17 ± 0.05	2.04 ± 0.23^a^ (55)	2.96 ± 0.22^a^ (132)	1.97 ± 0.27 (39)	1.90 ± 0.37^a^ (44)

Data are the means ± SEM of at least eight animals per group. Values in parentheses indicate the percentage of protection against thermal stimuli.

a *P < 0*.05 compared with the sodium bicarbonate (SB) control group,

b *P < 0*.05 compared with the peanut oil (PO) control group.

Adjuvant injection in rats can cause arthritic inflammation both at the site of injection (primary inflammation) and in the non-injected rear paw (secondary inflammation) (6). This model requires at least 14 days after induction for polyarthritis to occur. The onset of polyarthritis starts a secondary stage of the inflammatory response. Compared to the immunological primary inflammation, the secondary reaction usually occurs with a latency interval and is mediated by a delayed hypersensitivity reaction by T lymphocytes (1, 2 and 8). All doses of EE and F2 inhibited the secondary but not the primary immune inflammatory reactions. 

Determining the VSG is analogous to the determination of the analytical CRP test (15). VSG is directly related to the tendency of red blood cells to be stacked, and the elevation of plasma proteins (globulins and fibrinogen). As expected, in arthritic rats, ESR was increased compared with controls due to the inflammatory process (17). In arthritic rats, we found decreased haematocrit compared with normal control rats, but haemoglobin concentration was not changed. This suggests that at the time of study, anaemia was not present. Furthermore, body weight was not modified in arthritic rats compared to a normal control group during our study period.

Pro-inflammatory cytokines TNF-α, IL-1β, IL-6, and GM-CSF produced by CD4+ T cells and other cells such as macrophages and fibroblasts play an important role in the pathogenesis and progression of RA. IFN-γ and IL-18 are pro-inflammatory cytokines that are also involved in the progression of RA, but is thought that TNF-α is the major dominant modulator of pro-inflammatory cytokines (4, 5 and 25). Most systemic manifestations of inflammation are the result of the action of pro-inflammatory cytokines. For example, IL 1 and TNF-α produced by macrophages, act on the whole body to mobilize all available resources and combat harmful agents. Their action on the liver increases the synthesis of acute phase proteins such as the complement system and the CRP (15). In arthritic rats, CRP and RF were found in the serum, but not in normal control rats. This is due to thickening of the synovial membrane, the formation of creases and lymphocyte proliferation. These cells, which form lymphoid follicles, are responsible for the synthesis of rheumatoid factors and other immunoglobulins.

In this study, indomethacin, the EE and its fractions did not change the values of ESR, haematocrit, CRP or RF with respect to the SB control group because RA is a chronic degenerative inflammatory condition. The EE and its fractions decreased the concentrations of TNF-α, IL-6 and GM-CSF. It is interesting that *Eysenhardtia polystachya *decreased the serum concentration of TNF-α in arthritic rats because the blockade of this cytokine may have a more global effect on inflammation than the blockade of other cytokines present in high concentrations, such as IL-1 (5). These findings, along with the fact that all doses of the EE and F2 inhibited secondary but not primary immune inflammatory reactions, suggest that *Eysenhardtia polystachya *affects the cellular immune response, but further investigation is needed to better understand this effect. 

During the development of adjuvant-induced arthritis, acute periarticular inflammation characterized by infiltration of mononuclear cells into the synovial space is produced. Chronic arthritis then develops, including synovial hyperplasia and the destruction of periarticular bone and cartilage. Severe arthritis eventually causes bony ankylosis and leg deformities (6, 7 and 25). These characteristics were observed in the control group of arthritis. Furthermore, the EE and fractions partially inhibited the degradation of cartilage and bone, likely due to flavonoids.

Pain is a major symptom in patients with RA, and generally determines the effect of illness on the overall quality of life. Pain occurs as a disabling symptom for patients with RA and is considered as the most important consequence of the disease. According to Eddy and Leimback (20) and Koster *et al*. (19), thermal stimulation on the hot plate and writhing induced by acetic acid can be used to assess central and peripheral analgesic activity, respectively. Acetic acid increases AA metabolites such as prostaglandins (PG´s) by the COX pathway in peritoneal fluid. In particular, PGE_2_ increases serotonin, histamine, bradykinin and substance P, which excite pain nerve endings (26, 27). The nociceptive response caused by acetic acid is also dependent on release of certain cytokines such as TNF-α, IL-1β and IL-8 via macrophages and mast cells located in the peritoneal cavity (28). NSAIDs such as indomethacin decrease PG´s synthesis and prevent sensitization to nociceptors against pro-inflammatory mediators such as 5-hydroxytryptamine and bradykinin in peripheral tissues (22), thus interfering with the mechanism of transduction in primary afferent nociceptors (29). The EE and its fractions had a protective effect against chemical nociception to acetic acid similar to indomethacin. These results indicate that *Eysenhardtia polystachya *induces peripheral antinociceptive effects in inflammation pain (14), although its activity might also be related to suppression of the synthesis and/or release of endogenous algogenic substances such as PG´s (30), or to inhibition of the neurokinin 1 receptor (NK-1) of substance P, which is very abundant at the visceral level or its ability to decrease the concentration of pro-inflammatory interleukins.

The hot-plate test is based on a phasic stimulus of high intensity (30). The EE of *Eysenhardtia polystachya* and its fractions F1, F2 and F5 were effective in blocking nociception to thermal stimuli. These results indicate that the EE and its fractions have antinociceptive effects at the central level, probably by inhibiting the synthesis of PGs that facilitate the transmission of pain by afferents fibres to neurons in the dorsal horn (22) or by inhibition of transmission in the central level (30).

The anti-arthritic and antinociceptive mechanisms of the bark of *Eysenhardtia polystachya *are not yet known. Flavonoids could be responsible, but other chemicals could be equally important such as coumarins, quinones, saponins, and tannins. These phytochemicals have antiphlogistic and antinociceptive activities in other plant species (9, 30).

Despite the widespread use of herbal medicines, few scientific studies have been undertaken to ascertain the safety and efficacy of traditional remedies (21). The present investigation shows that the EE does not produce death, hence it can be concluded that *Eysenhardtia polystachya* is not orally toxic.

In conclusion, the ethanolic extract of *Eysenhardtia polystachya *bark and its rich-flavonoids fractions inhibited secondary inflammatory reactions in arthritic rats and delayed histopathological alterations of joint capsules. *Eysenhardtia polystachya* also decreased the serum levels of pro-inflammatory cytokines IL-6, TNF-α, and GM-CSF and showed antinociceptive activity at the peripheral and central levels, which suggests that this plant has an effect on the cellular immune response. These findings support the use of *Eysenhardtia polystachya* in Mexican folk medicine for treating rheumatoid arthritis and pain management. 

Importantly, fractions F2 and F5 of the ethanolic extract of *Eysenhardtia polystachya *bark showed the greatest anti-arthritic and antinociceptive activities in the animal models studied. Thus, chemical study of these fractions should continue to elucidate the components responsible for its biological activity.
